# After-Effects of Thixotropic Maneuvers on Chest Wall and Compartmental Operational Volumes of Healthy Subjects Using Optoelectronic Plethysmography

**DOI:** 10.3389/fphys.2019.01376

**Published:** 2019-11-01

**Authors:** Illia Nadinne Dantas Florentino Lima, Antonio Sarmento, Maria Clara Goes, Enrico Mazzuca, Antonella Lomauro, W. Darlene Reid, Andrea Aliverti, Guilherme Augusto De Freitas Fregonezi

**Affiliations:** ^1^PneumoCardioVascular Laboratory, Hospital Universitário Onofre Lopes, Empresa Brasileira de Serviços Hospitalares (EBSERH), Departamento de Fisioterapia, Universidade Federal do Rio Grande do Norte, Natal, Brazil; ^2^Laboratório de Inovação Tecnológica em Reabilitação, Departamento de Fisioterapia, Universidade Federal do Rio Grande do Norte, Natal, Brazil; ^3^Dipartimento di Elettronica, Informazione e Bioingegneria, Politecnico di Milano, Milan, Italy; ^4^Department of Physical Therapy, University of Toronto, Toronto, ON, Canada; ^5^Toronto Rehabilitation Institute, Interdepartmental Division of Critical Care Medicine, University of Toronto, Toronto, ON, Canada

**Keywords:** optoelectronic plethysmography, lung volume measurements, lung capacities, muscle thixotropy, respiratory muscles

## Abstract

The volumes assessed by optoelectronic plethysmography (OEP) and based on a three-compartmental model provide an accurate breath-by-breath index of expiratory and inspiratory (ribcage muscles and diaphragm) muscle length. Thus, after performing thixotropic maneuvers, OEP may also provide evidence regarding the history-dependent properties of these muscles. We studied the after-effects of different thixotropic conditionings on chest wall (CW) and compartmental operational volumes of 28 healthy subjects (25.5 ± 2.2 years, FVC_%pred_ 94.8 ± 5.5, and FEV_1__%pred_ 95.5 ± 8.9) using OEP. Conditionings were composed of inspiratory or expiratory contractions performed from total lung capacity (TLC) or residual volume (RV). The study protocol was composed of three consecutive contractions of the same maneuver, with 60 s of spontaneous breathing in between, and after-effects were studied in the first seven respiratory cycles of each contraction. Cumulative effects were also assessed by comparing the after-effects of each thixotropic maneuver. Inspiratory contractions performed from both TLC and RV acutely increased end-inspiratory (EIV) CW volumes (all *p* < 0.0001), mainly on both upper and lower ribcage compartments (i.e., non-diaphragmatic inspiratory muscles and diaphragm, respectively); while, expiratory contractions from RV decreased CW volumes (*p* < 0.0001) by reducing the upper ribcage and abdominal volumes (all *p* < 0.0001). The response of the thixotropic maneuvers did not present a cumulative effect. In healthy, the use of the three-compartmental model through OEP allows a detailed assessment of the diaphragm, inspiratory and expiratory muscle thixotropy. Furthermore, specific conditioning maneuvers led to thixotropy of the inspiratory ribcage, diaphragm, and expiratory muscles.

## Introduction

Skeletal muscle fibers possess complex biophysical properties that make the stiffness and resting tension at a given muscle length dependent on the previous history of movements and contractions. These history-dependent properties, referred in the biomedical literature to as thixotropy (Lakie et al., 1984; Hagbarth et al., 1985; Gregory et al., 1998), have several possible explanations. The most accepted is that proposed by Hill (1968) in which a fraction of the muscle fiber resting tension is generated by stable cross-bridges between actin and myosin filaments. In this sense, the short-range elastic component of the muscle fibers represents the mechanical effects of a small population of slowly cycling cross-bridges, and thixotropic behavior in relaxed muscles reflects a temporary reduction in the number of attached cross-bridges following a mechanical perturbation (Herbst, 1976).

According to Homma and Hagbarth (2000), the rib cage respiratory muscles also possess thixotropic properties. These authors proposed that the history-dependent passive properties of inspiratory muscles are an important component of expiration because expiratory movements need to stretch inspiratory muscles to reduce lung volume. Subsequently, Izumizaki et al. (2004) showed that the intensity and duration of muscle contraction, as well as lung volume and muscle relaxation affects thixotropy behavior. Moreover, the determinant of whether muscles became stiff or slack is muscle length when conditioned, thus lung volume at the time of the muscle contraction can be considered the most influential factor to end-expiratory volume (EEV) changes. Accordingly, a series of studies were performed in both healthy (Izumizaki et al., 2004, 2006c) and COPD patients (Izumizaki et al., 2006b, 2008b) in order to understand the after-effects of different thixotropic conditioning maneuvers on EEV, a measure of functional residual capacity.

The measurement of EEV can be performed indirectly from inspiratory capacity; however, this type of measurement does not follow the breath-by-breath volume variation and may be influenced by the subject’s cooperation. Using optoelectronic plethysmography (OEP), an accurate non-invasive method for measuring total chest wall (CW) volume and ventilation, these problems can be overcome, and changes of both tidal breath and EEV provide an assessment of inspiratory and expiratory muscle length (Dellaca et al., 2001; Aliverti et al., 2004; Wilkens et al., 2010; Fregonezi et al., 2018). In this context, and despite the existence of studies evaluating the presence of thixotropic properties on respiratory muscles especially based on new and more accurate technologies, the present study aimed to analyze the after-effects of different thixotropic conditionings (inspiratory or expiratory contractions) performed from higher (total lung capacity [TLC]) and lower (residual volume [RV]) volumes using OEP. As these after-effects are relatively fast in healthy subjects, the EEV and end-inspiratory (EIV) CW and compartmental volumes, were investigated for the first time on a breath-by-breath basis, thus the variation of both the EEV and EIV could be measured accurately in each single breath. Additionally, as compartmental volumes were analyzed based on a three-compartmental model (Aliverti et al., 2001), the after-effects of the thixotropic maneuvers on the diaphragm (acting on the lower ribcage) and expiratory muscles (acting on abdominal compartment) were also assessed.

Thus, despite the existence of studies evaluating the presence of thixotropic properties on respiratory muscles, the literature is still scarce, especially based on new and more accurate technologies. Accordingly, the present study aimed to analyze the after-effects of different thixotropic conditionings (inspiratory or expiratory contractions) performed from higher (TLC) and lower (RV) volumes using OEP. As these after-effects are relatively fast in healthy subjects, the EEV and EIV CW and compartmental volumes, were investigated for the first time on a breath-by-breath basis, thus the variation of both the EEV and EIV could be measured accurately in each single breath. Additionally, as compartmental volumes were analyzed based on a three-compartmental model (Aliverti et al., 2001), the after-effects of the thixotropic maneuvers on the diaphragm (acting on the lower ribcage) and expiratory muscles (acting on abdominal compartment) were also assessed.

## Materials and Methods

### Subjects

This is a cross-over study. Twenty-eight self-reported healthy subjects of both genders [14 males and 14 females; age 25.5 ± 2.2 years, with body mass index of 20.6 ± 2 kg/m^2^, forced vital capacity (FVC) of 4.7 ± 0.4 L and forced expiratory volume in the first second (FEV_1_) of 3.7 ± 0.5 L] with no history of smoking, without cardiac, neuromuscular or respiratory diseases were studied. Predicted values for pulmonary function were 94.8 ± 5.5% for FVC and 95.5 ± 8.9% for FEV_1_.

All subjects signed an informed consent form. The study was carried out in accordance with the recommendations of the confines of the World Medical Association Declaration of Helsinki for medical research using human participants and approved by the Research Ethics Committee (Federal University of Rio Grande do Norte, Brazil) under number 1.662.429/2016.

### Pulmonary Function

Pulmonary function was assessed by spirometry using a KoKo DigiDoser^®^ (Longmont, CO, United States) spirometer with subjects seated on a standard chair. Assessments were carried out following the acceptability and reproducibility criteria (American Thoracic Society [ATS]/European Respiratory Society [ERS], 2002) and the obtained values were compared to absolute and percentage of predicted spirometric values for the Brazilian population (Pereira et al., 2007).

### Chest Wall and Compartmental Volumes

The variation of the CW volume and its compartments [pulmonary ribcage (RCp), abdominal ribcage (RCa), and abdomen (AB)] were assessed using OEP (BTS Bioengineering, Milan, Italy). Six infrared TV cameras (three in front and three behind the subject), previously calibrated at a frequency of 60 frames s^–1^, were used to record the movement change of 89 retro-reflexive markers placed on the anterior and posterior surfaces of the thorax and abdomen of the subjects (Cala et al., 1996; Aliverti and Pedotti, 2003). A closed surface of the subject’s total CW was reconstructed by connecting the 3-dimensional coordinates of the markers, and the breath-by-breath volume enclosed by this surface was computed by means of an algorithm based on the Gauss theorem (Cala et al., 1996). This theorem allows the direct computation of the volume enclosed by the thoracoabdominal surface approximated by a closed mesh of triangles. An important feature of the OEP system is that it does not require any calibration based on maneuvers performed by the subject under analysis. In fact, CW volumes are obtained by the direct geometrical measurement of the 3-dimensional position of the points, and the accuracy does not depend upon the subject or the presence of any disease (D’Angelo et al., 2011).

From OEP, the end-inspiratory (EIV_CW_, EIV_RCp_, EIV_RCa_, and EIV_AB_) and EEV CW and compartmental volume variations (EEV_CW_, EEV_RCp_, EEV_RCa_, and EEV_AB_) were obtained during the protocol and included into data analysis. Additionally, as an altered breathing pattern may contribute to changes in operational volumes (O’Donnell et al., 2001), inspiratory (Ti), expiratory (Te), and total time of respiratory cycle (Ttot) as well as inspiratory and expiratory flow were also analyzed.

### Thixotropy Protocol

After the assessment of pulmonary function, the subjects were instructed to sit in a standard chair without back support and the retro-reflexive markers were positioned in the trunk surface of the subjects. Considering the history-dependency of skeletal muscle mechanical properties as well as to standardize the pre-test condition of the muscles before the experiments (Proske et al., 1993), after the explanation of each thixotropy maneuver, the subjects were instructed to remain seated and relaxed for 5 min before data acquisition.

The study protocol was composed of 1 min of quiet spontaneous breathing (QB) followed by a set of one of the following thixotropic maneuvers: Inspiratory contraction from TLC (ICo_TLC_); inspiratory contraction from RV (ICo_RV_); expiratory contraction from TLC (ECo_TLC_); or expiratory contraction from RV (ECo_RV_). All maneuvers were performed randomly with an interval of 15 min between them.

**FIGURE 1:**
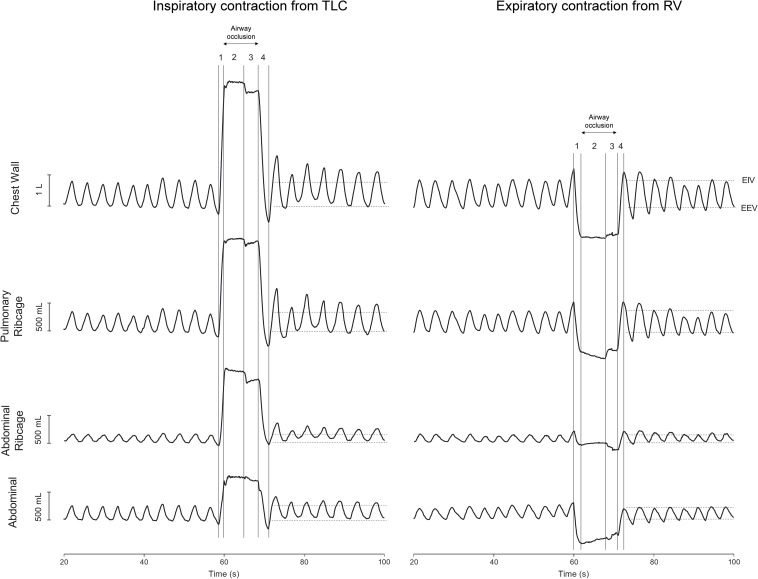
Representative traces of 10 cycles of quiet spontaneous breathing (QB) and the after-effects of inspiratory contraction from total lung capacity (TLC) and expiratory contraction from residual volume (RV) on the first seven cycles of chest wall and compartmental operational volumes. Original resting positions of both end-inspiratory (EIV) and end-expiratory (EEV) volumes obtained during 1 min of QB are indicated by horizontal dashed lines. **Left panel:** (1) inspiration to TLC, (2) sustained inspiratory effort, (3) voluntary muscle relaxation, and (4) passive expiration. **Right panel:** (1) expiration to RV, (2) sustained expiratory effort, (3) voluntary muscle relaxation, and (4) passive inspiration. Both records were obtained from the same subject. L, liters; mL, milliliters; s, seconds.

Each set was composed of three consecutive contractions of the same maneuver with 60 s of QB in between. Each maneuver consisted of the following steps (Figure 1): (1) maximal voluntary lung inflation (TLC) or deflation (RV); (2) five seconds of maximal sustained effort with the airway closed by a rigid mouthpiece shutter (breathed from a mouthpiece); (3) passive relaxation of 3 s; and (4) passive expiration or inspiration (Homma and Hagbarth, 2000; Izumizaki et al., 2004). After the passive relaxation, the shutter was manually reopened and the subject resumed breathing. During the whole protocol, the subjects were instructed to breathe with a mouthpiece coupled in the mouth.

### Data Analysis

The averaged of operational (EEV and EIV) CW and compartmental volumes, acquired during 1 min of QB before the first contraction, were defined as baseline levels. In each subject, the after-effects of the maneuvers on operational volumes were studied in detail immediately after each contraction (the first seven respiratory cycles) and compared to baseline. Here, shift values from baseline were signaled as changes in stiffness of respiratory muscles (Homma and Hagbarth, 2000; Izumizaki et al., 2004). Breathing pattern was also studied by analyzing inspiratory time (Ti), expiratory time (Te), and total time of respiratory cycle (Ttot) immediately after each thixotropic maneuver and compared with QB data obtained before the first contraction.

The cumulative effect of the maneuvers (i.e., significantly increases or decreases in CW and compartmental EIV or EEV) was also investigated by comparing each of the seven respiratory cycles after the second and third maneuvers with the after-effects of the first maneuver.

### Statistical Analysis

Anthropometric and spirometric data are shown as mean ± SD. Normality and data distribution were verified using Shapiro–Wilk test. Operational volumes, breathing pattern, and cumulative effects were compared using the ANOVA one-way for repeated measures or Friedman’s test followed by Bonferroni’s or Dunn’s *post hoc* tests, respectively, according to data normality.

Descriptive and inferential analyses were conducted using GraphPad Prism software, version 7.04 (GraphPad Inc., La Jolla, CA, United States). *P*-values lower than 0.05 (two-sided) were considered statistically significant.

## Results

### Inspiratory Contraction From TLC

**FIGURE 2:**
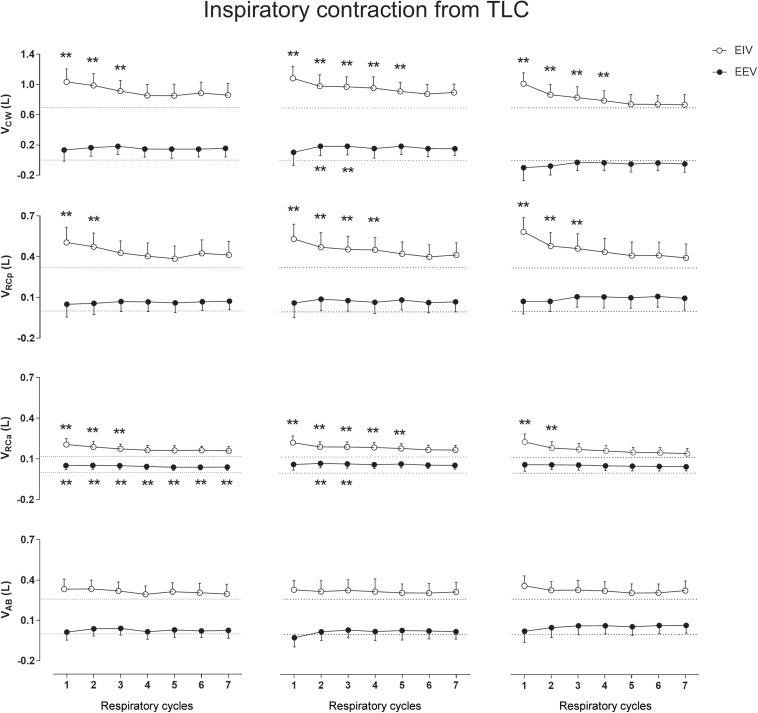
Aftereffects of inspiratory contraction maneuvers performed from total lung capacity (TLC) on end-inspiratory (EIV) and end-expiratory (EEV) volumes of chest wall (CW) and its compartments [pulmonary ribcage (RCp), abdominal ribcage (RCa), and abdominal (AB)]. Data of the first seven respiratory cycles (*x*-axis) are shown and were compared with mean values (mean of 60 s) of quiet breathing (dotted lines). **Left, middle**, and **right** columns shows these variations after the first, second, and third maneuvers, respectively. L, liters; ^∗∗^*p* < 0.0001 when compared with quiet breathing.

Figure 2 clearly shows that the inspiratory contraction maneuver performed from TLC changes significantly EIV rather than EEV. After the first maneuver, a significant increase in EIV_CW_, on the first three cycles (*p* < 0.0001, mean of 0.281 L, see Supplementary Material), was observed. A similar increase was found after the second maneuver, until the fifth respiratory cycle (*p* < 0.0001, mean of 0.282 L), as well as after the third maneuver until the fourth cycle (*p* < 0.0001, mean of 305 L). These increases were mainly due to significant changes in EIV_RCp_ (*p* < 0.0001) and EIV_RCa_ (*p* < 0.0001) after the first, second, and third maneuvers (see Supplementary  Material). Regarding EEV_CW_, no significant differences were observed when compared with QB values.

### Expiratory Contraction From TLC

**FIGURE 3:**
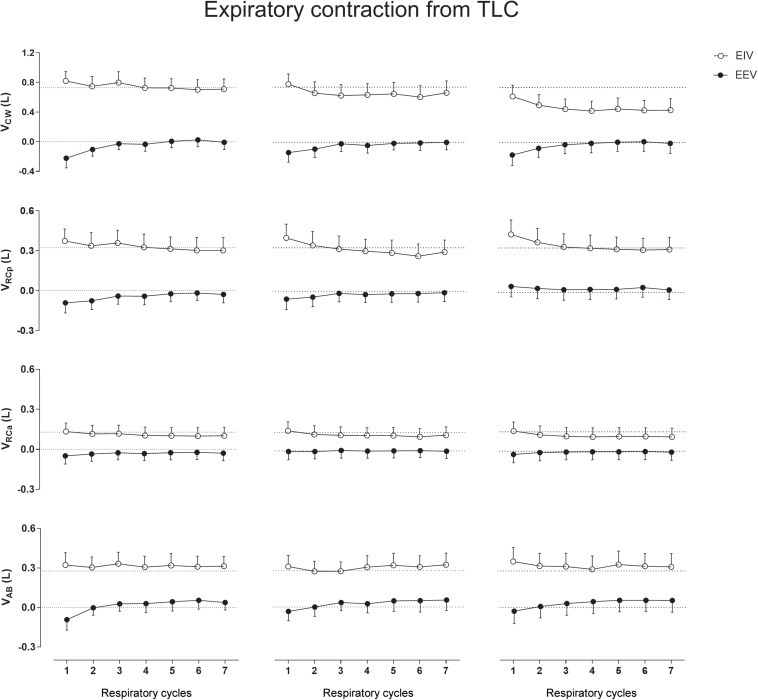
Aftereffects of expiratory contraction maneuvers performed from total lung capacity (TLC) on end-inspiratory (EIV) and end-expiratory (EEV) volumes of chest wall (CW) and its compartments [pulmonary ribcage (RCp), abdominal ribcage (RCa), and abdominal (AB)]. Data of the first seven respiratory cycles (*x*-axis) are shown and were compared with mean values (mean of 60 s) of quiet breathing (dotted lines). **Left, middle**, and **right** columns shows these variations after the first, second, and third maneuvers, respectively. L, liters.

No significant differences were observed in CW and compartmental EIV and EEV during this type of thixotropic contraction (Figure 3).

### Inspiratory Contraction From RV

**FIGURE 4:**
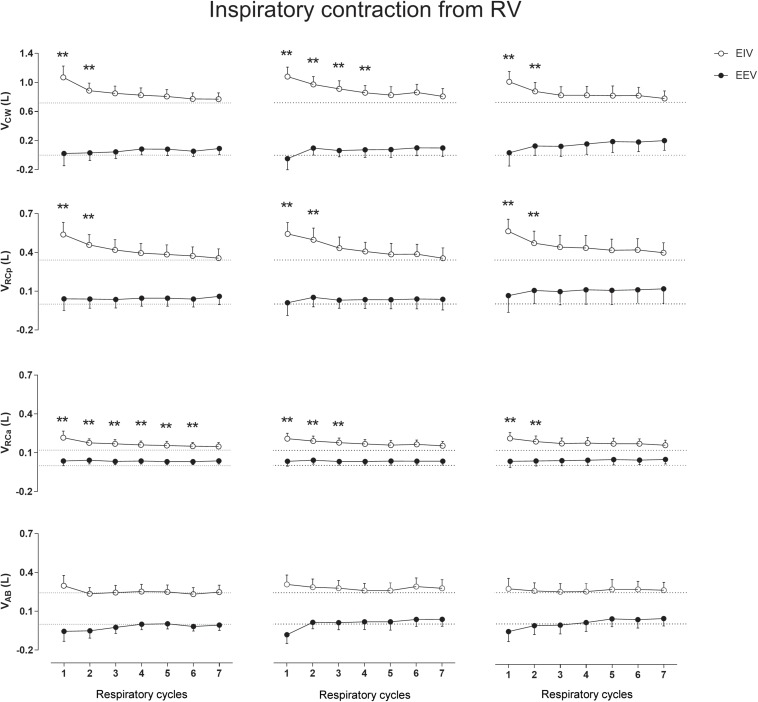
Aftereffects of inspiratory contraction maneuvers performed from residual volume (RV) on end-inspiratory (EIV) and end-expiratory (EEV) volumes of chest wall (CW) and its compartments [pulmonary ribcage (RCp), abdominal ribcage (RCa), and abdominal (AB)]. Data of the first seven respiratory cycles (*x*-axis) are shown and were compared with mean values (mean of 60 s) of quiet breathing (dotted lines). **Left, middle,** and **right** columns shows these variations after the first, second, and third maneuvers, respectively. L, liters; ^∗∗^*p* < 0.0001 when compared with quiet breathing.

Figure 4 shows that, when performed from RV, the inspiratory contractions lead to significant increases in EIV with no significant effects on EEV. Again, the RCp and RCa compartments were the main contributors to the significant increases in EIV_CW_ (*p* < 0.001) after each maneuver. Neither on RV nor on TLC the AB compartment participated significantly to changes in operational volumes.

### Expiratory Contraction From RV

**FIGURE 5:**
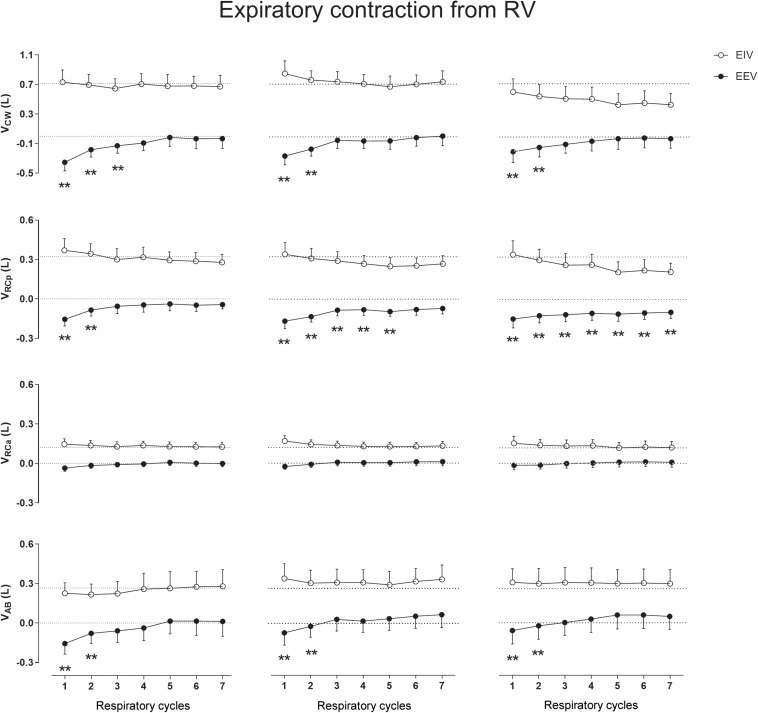
Aftereffects of expiratory contraction maneuvers performed from residual volume (RV) on end-inspiratory (EIV) and end-expiratory (EEV) volumes of chest wall (CW) and its compartments [pulmonary ribcage (RCp), abdominal ribcage (RCa), and abdominal (AB)]. Data of the first seven respiratory cycles (*x*-axis) are shown and were compared with mean values (mean of 60 s) of quiet breathing (dotted lines). **Left, middle**, and **right** columns shows these variations after the first, second, and third maneuvers, respectively. L, liters; ^∗∗^*p* < 0.0001 when compared with quiet breathing.

When compared with QB no significant differences were observed on CW and compartmental EIV after the EC_RV_ maneuver (Figure 5). On the other hand, the EC_RV_ was by far the most effective maneuver in decreasing EEV. Changes in EEV_CW_ were observed immediately after the first, second, and third expiratory contractions (mean of 0.220, 0.215, and 0.182 L, respectively) and were caused mainly by significant decreases in EEV_RCp_ (*p* < 0.0001) and EEV_AB_ (*p* < 0.0001) with no participation of the RCa compartment (see Supplementary Material and Zenodo Repository)^1^.

### Cumulative Effect Analysis

In all types of thixotropy contractions performed, no cumulative effects were observed between the three sets of maneuvers in both CW and compartmental EIV and EEV.

### Breathing Pattern

No significant differences in Ti, Te, and Ttot (see Supplementary Material) as well as inspiratory and expiratory flow (data not shown) were found after the conditioning maneuvers.

## Discussion

This is the first study to report the detailed after-effects of thixotropic maneuvers on the three-compartmental CW and compartmental operational volumes using OEP. The main findings of this study were that ICo_TLC_ and ICo_RV_ thixotropic conditionings affect equally the operational volumes of healthy subjects; i.e., increases EIV_CW_. More specifically, the after-effects of these two thixotropic conditionings were particularly pronounced on both RCp and RCa compartments. On the other hand, only the ECo_RV_ thixotropic conditioning was able to decrease EEV_RCp_ and EEV_AB_ with no influences on EIV.

The precise mechanisms responsible for the history-dependent thixotropic behavior of skeletal muscle fibers are still controversial (Axelson and Hagbarth, 2003). This history-dependent fraction of muscular resting tension may be partly explicable in terms of several hypotheses, namely, (1) cross-bridge formation between actin and myosin filaments, (2) the titin-actin, and (3) the Ca^2+^ interactions (Kellermayer et al., 1997) [surrounding passive tissues as well as pressure and viscosity of the intra- and inter-cellular fluids may also play a role. For review see Roberts (2016)]. According to Hill (1968), those cross-bridges formed in the resting muscle disrupt during stretch and only reform if the muscles are left undisturbed. Additionally, the reformation of those cross-bridges is not instantaneous, but may continue for several seconds and, during this interval, the resting tension gradually changes as a result of the built-up tension by newly formed cross-bridges (Campbell and Lakie, 1998). Regarding titin, a spring filament whose characteristic length and stiffness changes with the unfolding of molecular bonds (Kellermayer et al., 1997), its individual molecules are thixotropic by its nature since they display pronounced history-dependent viscoelastic behavior during an active stretch that may lead to exponential enhance in passive force production of the muscle (Kellermayer et al., 2001; Powers et al., 2014). Evidence also point out to the Ca^2+^ dependence in both cross-bridge (Campbell and Lakie, 1998; Campbell and Moss, 2000) and titin mechanisms (Labeit et al., 2003) (i.e., increasing the number of cross-bridges in the former, and changing the passive force levels in the latter) (Moss et al., 1976; Campbell and Moss, 2000; Tatsumi et al., 2001).

The results of previous studies (Homma and Hagbarth, 2000; Izumizaki et al., 2008a) showed that the after-effects of thixotropic conditionings on EEV were much more prominent when combining deep inspirations or expirations with strong isometric contractions of the respiratory muscles, being of minor importance the direction of the forced efforts (toward inspiration of expiration). This indicates that the after-effects of conditioning primarily depend on changes in passive properties attributable to cross-bridge kinetics. Although no significant effects were observed in our study when expiratory contractions were performed from TLC, a more detailed breath-by-breath analysis of the after-effects presented in Figure 3 shows a decrease in the first EEV_CW_ respiratory cycle (due to decreases in EEV_RCp_ and EEV_AB_) suggesting a pattern similar to that observed in Homma and Hagbarth’s (2000) study. On the other hand, after ICo_TLC_ and ECo_RV_ conditionings, it was observed that specific operational CW volumes change with the direction of contraction to the shortened muscle. At inflated lung volumes, the inspiratory muscles are shortened and expiratory muscles are lengthened, while the opposite occurs during lung deflation. In this sense, inspiratory and expiratory muscle stiffness could increase after ICo_TLC_ and ECo_RV_ conditioning, respectively, which could counteract the effects of the ensuing inspiratory muscle lengthening during lung deflation (Izumizaki et al., 2004) (and inflation, in case of ECo_RV_), thus increasing EIV_CW_ and EEV_CW_. Moreover, according to Homma and Hagbarth (2000), thixotropy of the antagonistic respiratory muscles also may occur and would work together with thixotropy of the contracted muscles. For example, if voluntary co-contraction of expiratory muscles occurs at an inflated lung position slackness will be introduced in the expiratory muscles favoring operational volume elevation.

The after-effects of both ICo_TLC_ and ICo_RV_ conditionings were clearly evidenced in the upper ribcage (RCp and RCa) and are in accordance with previous studies (Izumizaki et al., 2004, 2006a,b) performed using respiratory inductance plethysmography. Although measurements errors and artifactual EEV changes may be presented while using this system (American Thoracic Society [ATS]/European Respiratory Society [ERS], 2002), our results using OEP strongly supports the hypothesis that thixotropy of ribcage inspiratory muscles plays an important part in CW inflation (Homma and Hagbarth, 2000; Izumizaki et al., 2006c). In this context, and in the absence of such study using diaphragmatic fibers as well as assuming that diaphragm muscle is similar to limb muscles, the short-range elastic component of diaphragm fibers may also contribute to thixotropy of lengthening–shortening cycles and affect the mechanics of breathing (Sieck et al., 2013). Indeed, our findings suggest that specific conditioning maneuvers may result in diaphragm thixotropy behavior since significant increases in EIV_RCa_ were observed (Konno and Mead, 1967) during inspiratory contractions of both IC_TLC_ and IC_RV_ conditionings. These observations were feasible due to the high accuracy of the breath-by-breath OEP volume measurements as well as the use of the three-compartmental model (which provides an index of overall and compartmental inspiratory and expiratory muscle length and shortening), and may be of great interest in clinical rehabilitation for increasing lung volume in a population with pulmonary restrictive pattern.

Expiratory muscle activation following conditioning might contribute to thoracic-volume deflation, thus possibly reducing lung hyperinflation (Izumizaki et al., 2006b). In our study, it is possible that the AB compartment did not participate actively in the variation of operational volumes during ICo_TLC_, ICo_RV_, and ECo_TLC_ due to its function in stabilizing the CW (Loring and Mead, 1982). However, during the ECo_RV_ maneuver EEV_AB_ decreased significantly and, although changes in EEV_RCp_ accounted the most for the decrease in EEV_CW_ (see Supplementary Material) as also showed in a previous study (Homma and Hagbarth, 2000), these findings support the hypothesis that the expiratory muscles present thixotropy behavior (Izumizaki et al., 2006a, 2008a). Apart from being relatively fast (Izumizaki et al., 2006a), this transient effect may be of particularly importance in clinical settings, mainly in those COPD patients that present hyperinflation since contributes do abdominal deflation and consequently decrease in functional RV.

In our study using OEP, no lingering effects were observed. This result is in line with those reported by Campbell and Moss (2000) which showed that intrafilamentary movement of actively contracting muscles reduces the number of cross-bridges bound between the muscle filaments with a high cross-bridge reformation rate. As ribcage respiratory muscles are, by default, in constant contraction/relaxation activity during QB, the influence of both intrafilamentary movement of respiratory muscles and the time taken to the next thixotropy conditioning may have influenced the results. However, we believe that this was not the only factor affecting the results. Straining against a closed mouthpiece may also not affect the moles of gas in the lungs, but it will have considerable effects on the configuration of the thorax and also on lung volume via gas compression and rarefaction (which was not calculated due to lack of airway pressure measurements). This means that the various tissues of the thorax (ligaments, tendons, and connective in addition to muscle) will all be stretched, and since these tissues are all viscoelastic they will exhibit stress recovery afterward. The fact that expiratory contractions tended to affect EEV exclusively (Figures 3, 5), and vice versa for EIV (Figures 2, 4), supports the idea that viscoelastic structures were being stretched past their normal operating points for maximal expiration and inspiration, respectively.

It was also showed that in healthy subjects breathing pattern is not influenced by the conditioning maneuvers. The increases in EIV after ICo_TLC_ and ICo_RV_ could be simply a result of an increased inspiratory airflow since Ti remained constant. However, neither inspiratory nor expiratory flow was changed after these conditionings. Thus, the results support the assumption that breathing pattern is of minor importance to the thixotropic behavior of respiratory muscles in healthy subjects (Izumizaki et al., 2004).

A major limitation of our study was the lack of mouth pressure acquisition during the maneuvers. Nevertheless, a strong contraction was requested to all subjects during all procedures. The lack of EMG acquisition is also a major limitation since the findings regarding EIV could be a result of an increase in inspiratory muscle activation after a period of apnea. Additionally, it cannot be maintained that ribcage respiratory muscles alone were responsible for the after-effects alone. Activation of muscles other than those of the ribcage itself may have affected the results and, if these muscles also possess thixotropic properties, they may contribute to the observed after-effects (Homma and Hagbarth, 2000). Plastic properties of other structures in the CW and lung hysteresis may also play a role in changing the relaxed volume of the respiratory system. However, albeit we did not measure the after-effects on thoracoabdominal asynchrony, the use of the OEP and breath-by-breath analysis of the three-compartmental model of the CW clarify some questions raised by Izumizaki et al. (2006c) and provides new information about the influence of thixotropic conditionings on the muscles acting on RCp (non-diaphragmatic inspiratory muscles), RCa (diaphragm), and AB (expiratory muscles) compartments.

## Conclusion

The present study shows that both EIV and EEV CW and compartmental volumes change with different thixotropic conditionings. Moreover, the use of the three-compartmental model through OEP allowed the demonstration of the diaphragm and expiratory muscle thixotropy evidence in healthy subjects. The results can also be of great interest in clinical rehabilitation since specific thixotropic conditionings may be useful in reducing functional residual capacity as well as increase EIV.

## Data Availability Statement

All datasets generated for this study are included in the article/Supplementary  Material.

## Ethics Statement

The studies involving human participants were reviewed and approved by the Research Ethics Committee (Federal University of Rio Grande do Norte, Brazil) under number 1.662.429/2016. The patients/participants provided their written informed consent to participate in this study.

## Author Contributions

IL, AA, and GF designed and supervised the study. IL, AS, MG, and AL collected the data. IL, AS, AA, EM, and AL analyzed and interpreted the data. IL wrote the article. WR, AA, and GF revised the article.

## Conflict of Interest

The authors declare that the research was conducted in the absence of any commercial or financial relationships that could be construed as a potential conflict of interest.

## Footnotes

1 http://doi.org/10.5281/zenodo.3402458
