# Supplementary material for: After-Effects of Thixotropic Maneuvers on Chest Wall and Compartmental Operational Volumes of Healthy Subjects Using Optoelectronic Plethysmography
Source: Front Physiol. 2019 Nov 1;10:1376. doi: 10.3389/fphys.2019.01376 (PMC6838213; doi:10.3389/fphys.2019.01376)
Supplement: Supplementary file 1 [file Table_1.DOCX]

**Supplementary table 1**. Effects of **inspiratory contraction from total lung capacity** on inspiratory time (Ti), expiratory time (Te) and total time of respiratory cycle (Ttot), end-inspiratory (EIV) and end-expiratory (EEV) chest wall (CW) and compartmental (pulmonary ribcage [RCp]; abdominal ribcage [RCa] and abdominal [Ab]) volume changes (∆) after first (A), second (B) and third (C) maneuvers.

**A)**

|  | **1º cycle** | **2º cycle** | **3º cycle** | **4º cycle** | **5º cycle** | **6º cycle** | **7º cycle** |
| --- | --- | --- | --- | --- | --- | --- | --- |
| **∆EIV_RCp_** | **0.178 ± 0.281** | **0.147 ± 0.255** | 0.101 ±0.224 | 0.077 ± 0.246 | 0.058 ±0.236 | 0.098 ± 0.250 | 0.085 ± 0.254 |
| **∆EIV_RCa_** | **0.087 ± 0.107** | **0.069 ±0.102** | **0.055 ± 0.085** | 0.045 ± 0.088 | 0.043 ± 0.089 | 0.044 ±0.073 | 0.040 ± 0.080 |
| **∆EIV_AB_** | 0.072 ±0.189 | 0.074 ± 0.164 | 0.059 ± 0.166 | 0.035 ±0.152 | 0.053 ± 0.168 | 0.046 ± 0.176 | 0.037 ± 0.179 |
| **∆EIV_CW_** | **0.338 ± 0.439** | **0.290 ± 0.394** | **0.215 ± 0.356** | 0.157 ± 0.368 | 0.155 ± 0.387 | 0.189 ± 0.365 | 0.163 ± 0.385 |
| **∆EEV_RCp_** | 0.049 ± 0.240 | 0.056 ± 0.211 | 0.068 ± 0.184 | 0.066 ± 0.182 | 0.059 ± 0.183 | 0.067 ± 0.160 | 0.071 ± 0.155 |
| **∆EEV_RCa_** | 0.052 ± 0.077 | 0.052 ± 0.072 | 0.051 ± 0.066 | 0.044 ± 0.062 | 0.039 ± 0.058 | 0.039 ± 0.050 | 0.040 ± 0.058 |
| **∆EEV_AB_** | 0.014 ± 0.153 | 0.037 ± 0.134 | 0.041 ± 0.127 | 0.016 ± 0.139 | 0.029 ± 0.139 | 0.022 ± 0.120 | 0.026 ± 0.148 |
| **∆EEV_CW_** | 0.123 ± 0.362 | 0.154 ± 0.277 | 0.172 ± 0.271 | 0.136 ± 0.262 | 0.136 ± 0.298 | 0.135 ± 0.253 | 0.146 ± 0.283 |
| **Ti (s)** | 1,881 ± 0,784 | 1,961 ± 0,765 | 1,870 ± 0,652 | 1,865 ± 0,565 | 1,940 ± 0,621 | 1,946 ± 0,616 | 1,772 ± 0,538 |
| **Te (s)** | 2,380 ± 0,853 | 2,316 ± 0,950 | 2,463 ± 0,729 | 2,443 ± 0,952 | 2,441 ± 0,944 | 2,427 ± 0,802 | 2,178 ± 0,740 |
| **Ttot(s)** | 4,270 ± 1,459 | 4,279 ± 1,514 | 4,370 ± 1,211 | 4,354 ± 1,390 | 4,397 ± 1,398 | 4,384 ± 1,246 | 3,967 ± 1,216 |

Data shown as mean ± standard deviation. Dark black numbers represent significant differences when compared with quiet breathing.

**B)**

|  | **1º cycle** | **2º cycle** | **3º cycle** | **4º cycle** | **5º cycle** | **6º cycle** | **7º cycle** |
| --- | --- | --- | --- | --- | --- | --- | --- |
| **∆EIV_RCp_** | **0.217 ± 0.275** | **0.156 ±0.272** | **0.140 ± 0.247** | **0.136 ± 0.236** | 0.108 ± 0.219 | 0.086 ± 0.227 | 0.099 ± 0.228 |
| **∆EIV_RCa_** | **0.102 ± 0.124** | **0.070 ± 0.097** | **0.069 ± 0.095** | **0.066 ± 0.096** | **0.059 ±0.090** | 0.049 ± 0.087 | 0.047 ± 0.088 |
| **∆EIV_AB_** | 0.067 ± 0.178 | 0.055 ±0.203 | 0.063 ± 0.199 | 0.054 ± 0.238 | 0.045 ± 0.165 | 0.044 ± 0.178 | 0.052 ± 0.174 |
| **∆EIV_CW_** | **0.387 ± 0.390** | **0.282 ± 0.382** | **0.273 ± 0.334** | **0.257 ± 0.376** | **0.213 ± 0.302** | 0.180 ± 0.321 | 0.200 ± 0.279 |
| **∆EEV_RCp_** | 0.061 ± 0.272 | 0.089 ± 0.211 | 0.078 ± 0.195 | 0.066 ± 0.213 | 0.083 ± 0.188 | 0.063 ± 0.189 | 0.069 ± 0.190 |
| **∆EEV_RCa_** | 0.055 ± 0.107 | 0.062 ± 0.078 | 0.059 ± 0.077 | 0.053 ± 0.072 | 0.058 ± 0.074 | 0.050 ± 0.065 | 0.048 ± 0.070 |
| **∆EEV_AB_** | -0.007 ± 0.173 | 0.036 ± 0.165 | 0.049 ± 0.146 | 0.037 ± 0.171 | 0.045 ± 0.177 | 0.042 ± 0.149 | 0.037 ± 0.138 |
| **∆EEV_CW_** | 0.108 ± 0.440 | **0.188 ± 0.312** | **0.187 ± 0.286** | 0.157 ± 0.318 | 0.187 ± 0.281 | 0.156 ± 0.273 | 0.154 ± 0.226 |
| **Ti (s)** | 2,202 ± 0,661 | 1,863 ± 0,520 | 2,298 ± 0,982 | 2,170 ± 1,32 | 1,823 ± 0,566 | 1,760 ± 0,530 | 1,860 ± 0,540 |
| **Te (s)** | 2,580 ± 1,175 | 2,341 ± 0,838 | 2,394 ± 0,904 | 2,337 ± 0,764 | 2,286 ± 0,876 | 2,202 ± 0,734 | 2,314 ± 0,863 |
| **Ttot(s)** | 4,803 ±1,716 | 4,244 ±1,202 | 4,705 ±1,673 | 4,553 ± 1,926 | 4,123 ± 1,308 | 3,989 ± 1,217 | 4,195 ± 1,341 |

Data shown as mean ± standard deviation. Dark black numbers represent significant differences when compared with quiet breathing.

**C)**

|  | **1º cycle** | **2º cycle** | **3º cycle** | **4º cycle** | **5º cycle** | **6º cycle** | **7º cycle** |
| --- | --- | --- | --- | --- | --- | --- | --- |
| **∆EIV_RCp_** | **0.263 ± 0.256** | **0.159 ± 0.243** | **0.140 ± 0.269** | 0.114 ± 0.251 | 0.088 ± 0.250 | 0.089 ± 0.242 | 0.072 ± 0.255 |
| **∆EIV_RCa_** | **0.113 ± 0.149** | **0.068 ± 0.118** | 0.058 ± 0.110 | 0.047 ± 0.107 | 0.036 ± 0.094 | 0.034 ± 0.095 | 0.028 ± 0.092 |
| **∆EIV_AB_** | 0.105 ± 0.188 | 0.071 ±0.165 | 0.073 ± 0.182 | 0.067 ± 0.171 | 0.051 ± 0.172 | 0.053 ± 0.166 | 0.069 ± 0.179 |
| **∆EIV_CW_** | **0.444 ± 0.359** | **0.298 ± 0.334** | **0.260 ± 0.354** | **0.220 ± 0.321** | 0.171 ±0.318 | 0.170 ± 0.300 | 0.165 ± 0.337 |
| **∆EEV_RCp_** | 0.063 ± 0.235 | 0.062 ± 0.189 | 0.097 ± 0.194 | 0.096 ± 0.208 | 0.089 ± 0.196 | 0.099 ± 0.199 | 0.086 ± 0.224 |
| **∆EEV_RCa_** | 0.054 ± 0.122 | 0.053 ± 0.089 | 0.052 ± 0.091 | 0.046 ± 0.092 | 0.043 ± 0.088 | 0.041 ± 0.086 | 0.039 ± 0.078 |
| **∆EEV_AB_** | 0.008 ± 0.211 | 0.034 ± 0.185 | 0.047 ± 0.166 | 0.049 ± 0.151 | 0.041 ± 0.161 | 0.050 ± 0.147 | 0.050 ± 0.143 |
| **∆EEV_CW_** | 0.125 ± 0.436 | 0.148 ± 0.299 | 0.194 ± 0.272 | 0.190 ± 0.266 | 0.174 ± 0.269 | 0.188 ± 0.260 | 0.175 ± 0.280 |
| **Ti (s)** | 2,190 ± 0,842 | 1,998 ± 0,717 | 2,042 ± 0,795 | 1,846 ± 0,611 | 2,004 ± 0,754 | 1,856 ± 0,584 | 2,068 ± 0,810 |
| **Te (s)** | 2,469 ± 0,821 | 2,322 ± 0,849 | 2,269 ± 0,869 | 2,290 ± 0,775 | 2,528 ± 1,260 | 2,188 ± 0,779 | 2,098 ± 0,717 |
| **Ttot(s)** | 4,691 ± 1,528 | 4,318 ± 1,440 | 4,267 ± 1,525 | 4,153 ± 1,310 | 4,511 ± 1,700 | 4,058 ± 1,256 | 4,191 ± 1,393 |

Data shown as mean ± standard deviation. Dark black numbers represent significant differences when compared with quiet breathing.

**Supplementary table 2**. Effects of **expiratory contraction from total lung capacity** on inspiratory time (Ti), expiratory time (Te) and total time of respiratory cycle (Ttot), end-inspiratory (EIV) and end-expiratory (EEV) chest wall (CW) and compartmental (pulmonary ribcage [RCp]; abdominal ribcage [RCa] and abdominal [Ab]) volume changes (∆) after first (A), second (B) and third (C) maneuvers.

**A)**

|  | **1º cycle** | **2º cycle** | **3º cycle** | **4º cycle** | **5º cycle** | **6º cycle** | **7º cycle** |
| --- | --- | --- | --- | --- | --- | --- | --- |
| **∆EIV_RCp_** | 0.047 ± 0.231 | 0.011 ± 0.249 | 0.032 ± 0.247 | 0.009 ± 0.251 | -0.011 ± 0.227 | -0.023 ± 0.248 | -0.023 ± 0.243 |
| **∆EIV_RCa_** | 0.003 ± 0.162 | -0.014 ± 0.161 | -0.012 ± 0.161 | -0.026 ± 0.158 | -0.028 ± 0.154 | -0.030 ± 0.159 | -0.028 ± 0.162 |
| **∆EIV_AB_** | 0.038 ± 0.231 | 0.019 ± 0.196 | 0.047 ± 0.219 | 0.022 ± 0.208 | 0.034 ± 0.223 | 0.025 ± 0.196 | 0.029 ± 0.182 |
| **∆EIV_CW_** | 0.089 ± 0.320 | 0.017 ±0.337 | 0.067 ±0.369 | -0.003 ± 0.339 | -0.005 ± 0.315 | -0.028 ± 0.340 | -0.021 ± 0.350 |
| **∆EEV_RCp_** | -0.091 ± 0.191 | -0.076 ± 0.167 | -0.041 ± 0.152 | -0.041 ± 0.163 | -0.023 ± 0.149 | -0.017 ± 0.145 | -0.028 ± 0.159 |
| **∆EEV_RCa_** | -0.043 ± 0.155 | -0.029 ± 0.140 | -0.020 ± 0.135 | -0.026 ± 0.133 | -0.019 ± 0.136 | -0.016 ± 0.139 | -0.023 ± 0.144 |
| **∆EEV_AB_** | -0.092 ± 0.202 | -0.001 ± 0.145 | 0.028 ± 0.143 | 0.029 ± 0.175 | 0.043 ± 0.176 | 0.054 ± 0.166 | 0.038 ± 0.144 |
| **∆EEV_CW_** | -0.227 ± 0.330 | -0.108 ± 0.223 | -0.032 ± 0.199 | -0.039 ± 0.239 | 0.009 ± 0.220 | 0.020 ± 0.233 | -0.012 ± 0.247 |
| **Ti (s)** | 1,979 ± 0,566 | 1,960 ± 0,659 | 2,016 ± 0,891 | 1,916 ± 0,815 | 2,079 ± 1,006 | 1,972 ± 0,672 | 1,849 ± 0,698 |
| **Te (s)** | 2,291 ± 0,616 | 2,933 ± 2,189 | 2,404 ± 1,299 | 2,082 ± 0,582 | 2,272 ± 0,866 | 2,412 ± 0,714 | 2,305 ± 0,913 |
| **Ttot(s)** | 4,270 ± 1,087 | 4,893 ± 2,269 | 4,419 ± 1,733 | 3,998 ± 1,220 | 4,351 ± 1,753 | 4,384 ± 1,312 | 4,154 ± 1,402 |

Data shown as mean ± standard deviation. Dark black numbers represent significant differences when compared with quiet breathing

**B)**

|  | **1º cycle** | **2º cycle** | **3º cycle** | **4º cycle** | **5º cycle** | **6º cycle** | **7º cycle** |
| --- | --- | --- | --- | --- | --- | --- | --- |
| **∆EIV_RCp_** | 0.079 ± 0.262 | 0.023 ± 0.262 | -0.003 ± 0.248 | -0.019 ± 0.227 | -0.032 ± 0.243 | -0.056 ± 0.230 | -0.027 ± 0.229 |
| **∆EIV_RCa_** | 0.016 ± 0.171 | -0.011 ±0.166 | -0.017 ±0.158 | -0.019 ±0.149 | -0.019 ± 0.153 | -0.028 ±0.155 | -0.016 ±0.157 |
| **∆EIV_AB_** | 0.029 ± 0.210 | -0.007 ± 0.192 | -0.006 ± 0.178 | 0.023 ± 0.220 | 0.038 ± 0.227 | 0.025 ± 0.214 | 0.041 ±0.221 |
| **∆EIV_CW_** | 0.078 ± 0.349 | -0.042 ±0.385 | -0.074 ± 0.372 | -0.065 ± 0.379 | -0.052 ±0.392 | -0.095 ±0.387 | -0.038 ± 0.403 |
| **∆EEV_RCp_** | -0.061 ± 0.203 | -0.046 ± 0.182 | -0.018 ± 0.162 | -0.026 ± 0.152 | -0.021 ± 0.163 | -0.018 ± 0.166 | -0.013 ± 0.167 |
| **∆EEV_RCa_** | -0.015 ± 0.156 | -0.015 ± 0.141 | -0.006 ± 0.147 | -0.011 ± 0.136 | -0.010 ± 0.133 | -0.008 ± 0.131 | -0.012 ± 0.136 |
| **∆EEV_AB_** | -0.037 ± 0.178 | -0.004 ± 0.178 | 0.030 ± 0.159 | 0.021 ± 0.174 | 0.043 ± 0.200 | 0.044 ± 0.219 | 0.049 ± 0.200 |
| **∆EEV_CW_** | -0.113 ± 0.327 | -0.065 ± 0.287 | 0.005 ± 0.266 | -0.017 ± 0.258 | 0.011 ± 0.225 | 0.017 ± 0.255 | 0.024 ± 0.252 |
| **Ti (s)** | 2,212 ± 1,061 | 1,839 ± 0,451 | 1,903 ± 0,634 | 1,939 ± 0,675 | 2,014 ± 0,819 | 1,861 ± 0,474 | 1,840 ± 0,611 |
| **Te (s)** | 2,681 ± 0,948 | 2,398 ± 0,835 | 2,435 ± 0,806 | 2,226 ± 0,706 | 2,382 ± 0,686 | 2,030 ± 0,638 | 2,312 ± 0,624 |
| **Ttot(s)** | 4,893 ± 1,829 | 4,237 ± 0,970 | 4,339 ± 1,348 | 4,165 ± 1,241 | 4,397 ± 1,426 | 3,891 ± 1,011 | 4,153 ± 1,149 |

Data shown as mean ± standard deviation. Dark black numbers represent significant differences when compared with quiet breathing.

**C)**

|  | **1º cycle** | **2º cycle** | **3º cycle** | **4º cycle** | **5º cycle** | **6º cycle** | **7º cycle** |
| --- | --- | --- | --- | --- | --- | --- | --- |
| **∆EIV_RCp_** | 0.097 ± 0.276 | 0.037 ± 0.266 | 0.002 ±0.251 | -0.007 ±0.252 | -0.015 ±0.233 | -0.020 ± 0.225 | -0.016 ± 0.235 |
| **∆EIV_RCa_** | 0.015 ± 0.171 | -0.013 ± 0.168 | -0.024 ± 0.164 | -0.028 ± 0.170 | -0.025 ± 0.166 | -0.025 ± 0.164 | -0.028 ± 0.167 |
| **∆EIV_AB_** | 0.072 ± 0.270 | 0.038 ± 0.243 | 0.034 ± 0.256 | 0.013 ± 0.255 | 0.049 ± 0.257 | 0.037 ± 0.242 | 0.032 ± 0.254 |
| **∆EIV_CW_** | 0.223 ± 0.386 | 0.105 ±0.364 | 0.054 ± 0.341 | 0.028 ± 0.336 | 0.055 ±0.376 | 0.037 ± 0.336 | 0.039 ± 0.394 |
| **∆EEV_RCp_** | 0.024 ± 0.198 | 0.009 ± 0.197 | -0.009 ± 0.201 | 0.001 ± 0.194 | 0.002 ± 0.184 | 0.016 ± 0.188 | -0.001 ± 0.185 |
| **∆EEV_RCa_** | -0.035 ± 0.160 | -0.022 ± 0.156 | -0.017 ± 0.148 | -0.016 ± 0.151 | -0.016 ± 0.152 | -0.014 ± 0.154 | -0.018 ± 0.156 |
| **∆EEV_AB_** | -0.035 ± 0.240 | 0.009 ± 0.217 | 0.022 ± 0.223 | 0.036 ± 0.230 | 0.046 ± 0.220 | 0.046 ± 0.216 | 0.046 ± 0.227 |
| **∆EEV_CW_** | -0.153 ± 0.355 | -0.062 ± 0.313 | -0.014 ±0.299 | 0.003 ± 0.322 | 0.019 ±0.312 | 0.026 ± 0.338 | 0.004 ± 0.345 |
| **Ti (s)** | 1,998 ± 0,766 | 2,005 ± 0,788 | 2,033 ± 0,670 | 1,937 ± 0,684 | 2,000 ± 0,653 | 1,856 ± 0,600 | 1,960 ± 0,662 |
| **Te (s)** | 2,360 ± 0,817 | 2,105 ± 0,671 | 2,212 ± 0,632 | 2,198 ± 0,689 | 2,219 ± 0,738 | 2,325 ± 0,903 | 2,100 ± 0,644 |
| **Ttot(s)** | 4,411 ± 1,333 | 4,209 ± 1,508 | 4,377 ± 1,252 | 4,133 ± 1,203 | 4,253 ± 1,333 | 4,226 ± 1,326 | 4,361 ± 1,367 |

Data shown as mean ± standard deviation. Dark black numbers represent significant differences when compared with quiet breathing.

**Supplementary table 3**. Effects of **inspiratory contraction from residual volume** on inspiratory time (Ti), expiratory time (Te) and total time of respiratory cycle (Ttot), end-inspiratory (EIV) and end-expiratory (EEV) chest wall (CW) and compartmental (pulmonary ribcage [RCp]; abdominal ribcage [RCa] and abdominal [Ab]) volume changes (∆) after first (A), second (B) and third (C) maneuvers.

**A)**

|  | **1º cycle** | **2º cycle** | **3º cycle** | **4º cycle** | **5º cycle** | **6º cycle** | **7º cycle** |
| --- | --- | --- | --- | --- | --- | --- | --- |
| **∆EIV_RCp_** | **0.198 ± 0.238** | **0.118 ± 0.199** | 0.078 ± 0.201 | 0.054 ± 0.184 | 0.044 ± 0.185 | 0.032 ± 0.175 | 0.015 ± 0.181 |
| **∆EIV_RCa_** | **0.101 ± 0.127** | **0.060 ± 0.078** | **0.053 ±0.080** | **0.044 ± 0.076** | **0.039 ± 0.081** | **0.034 ± 0.072** | 0.031 ± 0.076 |
| **∆EIV_AB_** | 0.051 ± 0.202 | -0.010 ± 0.122 | -0.001 ±0.139 | 0.006 ±0.140 | 0.004 ± 0.133 | -0.013 ± 0.129 | 0.002 ± 0.133 |
| **∆EIV_CW_** | **0.350 ± 0.393** | **0.167 ±0.260** | 0.130 ± 0.248 | 0.105 ±0.249 | 0.087 ±0.236 | 0.053 ± 0.208 | 0.050 ± 0.218 |
| **∆EEV_RCp_** | 0.041 ± 0.227 | 0.039 ± 0.178 | 0.036 ± 0.166 | 0.046 ±0.155 | 0.046 ±0.154 | 0.039 ± 0.157 | 0.060 ± 0.160 |
| **∆EEV_RCa_** | 0.037 ± 0.093 | 0.042 ± 0.071 | 0.033 ±0.063 | 0.037 ± 0.059 | 0.032 ± 0.060 | 0.031 ± 0.058 | 0.037 ± 0.060 |
| **∆EEV_AB_** | -0.054 ± 0.195 | -0.050 ± 0.141 | -0.023 ± 0.119 | 0.009 ± 0.105 | 0.003 ± 0.101 | -0.017 ±0.087 | -0.006 ± 0.102 |
| **∆EEV_CW_** | 0.023 ± 0.429 | 0.031 ± 0.265 | 0.045 ± 0.234 | 0.083 ±0.203 | 0.081 ±0.221 | 0.054 ± 0.184 | 0.091 ± 0.213 |
| **Ti (s)** | 1,896 ± 0,609 | 2,100 ± 0,858 | 2,239 ± 0,781 | 2,012 ± 0,691 | 1,909 ± 0,731 | 1,956 ± 0,974 | 1,879 ± 0,577 |
| **Te (s)** | 2,694 ± 1,059 | 2,367 ± 0,807 | 2,335 ± 0,768 | 2,520 ± 0,877 | 2,702 ± 0,975 | 2,200 ± 0,690 | 2,386 ± 0,806 |
| **Ttot(s)** | 4,823 ± 1,671 | 4,658 ± 1,411 | 4,365 ± 1,356 | 4,512 ± 1,542 | 4,690 ± 1,657 | 4,153 ± 1,201 | 4,289 ± 1,445 |

Data shown as mean ± standard deviation. Dark black numbers represent significant differences when compared with quiet breathing.

**B)**

|  | **1º cycle** | **2º cycle** | **3º cycle** | **4º cycle** | **5º cycle** | **6º cycle** | **7º cycle** |
| --- | --- | --- | --- | --- | --- | --- | --- |
| **∆EIV_RCp_** | **0.209 ± 0.223** | **0.163 ± 0.228** | 0.098 ± 0.218 | 0.073 ± 0.178 | 0.050 ± 0.214 | 0.051 ± 0.195 | 0.020 ± 0.201 |
| **∆EIV_RCa_** | **0.096 ± 0.106** | **0.078 ±0.096** | **0.065 ±0.090** | 0.055 ±0.086 | 0.046 ±0.091 | 0.051 ± 0.084 | 0.040 ± 0.084 |
| **∆EIV_AB_** | 0.064 ± 0.184 | 0.043 ±0.159 | 0.035 ±0.150 | 0.017 ±0.139 | 0.017 ± 0.149 | 0.047 ± 0.169 | 0.034 ± 0.169 |
| **∆EIV_CW_** | **0.369 ± 0.326** | **0.260 ± 0.271** | **0.199 ± 0.283** | **0.146 ± 0.248** | 0.114 ±0.292 | 0.151 ±0.278 | 0.095 ± 0.273 |
| **∆EEV_RCp_** | 0.018 ± 0.251 | 0.059 ± 0.184 | 0.038 ± 0.159 | 0.041 ±0.172 | 0.041 ±0.177 | 0.046 ± 0.192 | 0.044 ± 0.210 |
| **∆EEV_RCa_** | 0.042 ± 0.096 | 0.050 ± 0.075 | 0.040 ± 0.070 | 0.040 ± 0.067 | 0.043 ± 0.076 | 0.043 ± 0.074 | 0.042 ± 0.075 |
| **∆EEV_AB_** | -0.103 ± 0.172 | -0.007 ± 0.127 | -0.010 ± 0.139 | -0.004 ± 0.150 | -0.003 ±0.161 | 0.014 ± 0.137 | 0.015 ± 0.133 |
| **∆EEV_CW_** | -0.043 ± 0.387 | 0.101 ± 0.243 | 0.067 ± 0.218 | 0.077 ± 0.262 | 0.080 ± 0.269 | 0.104 ± 0.271 | 0.102 ± 0.290 |
| **Ti (s)** | 2,130 ± 0,769 | 2,123 ± 0,761 | 2,061 ± 0,661 | 1,888 ± 0,492 | 1,970 ± 0,622 | 2,253 ± 1,071 | 1,918 ± 0,646 |
| **Te (s)** | 2,473 ± 0,891 | 2,531 ± 0,837 | 2,476 ± 0,853 | 2,357 ± 0,825 | 2,500 ± 1,042 | 2,482 ± 0,897 | 2,425 ± 0,731 |
| **Ttot(s)** | 4,618 ± 1,555 | 4,649 ± 1,452 | 4,549 ± 1,450 | 4,233 ± 1,193 | 4,447 ± 1,400 | 4,735 ± 1,634 | 4,316 ± 1,375 |

Data shown as mean ± standard deviation. Dark black numbers represent significant differences when compared with quiet breathing.

**C)**

|  | **1º cycle** | **2º cycle** | **3º cycle** | **4º cycle** | **5º cycle** | **6º cycle** | **7º cycle** |
| --- | --- | --- | --- | --- | --- | --- | --- |
| **∆EIV_RCp_** | **0.216 ± 0.235** | **0.124 ± 0.233** | 0.093 ± 0.231 | 0.087 ± 0.244 | 0.069 ± 0.218 | 0.072 ± 0.218 | 0.050 ± 0.195 |
| **∆EIV_RCa_** | **0.098 ± 0.118** | **0.073 ± 0.110** | 0.058 ± 0.107 | 0.062 ± 0.107 | 0.057 ± 0.108 | 0.056 ± 0.096 | 0.045 ± 0.099 |
| **∆EIV_AB_** | 0.041 ± 0.205 | 0.025 ± 0.158 | 0.018 ± 0.159 | 0.020 ± 0.160 | 0.037 ± 0.191 | 0.037 ±0.158 | 0.030 ± 0.154 |
| **∆EIV_CW_** | **0.356 ± 0.356** | **0.223 ± 0.310** | 0.170 ± 0.293 | 0.170 ± 0.311 | 0.163 ± 0.338 | 0.166 ±0.288 | 0.126 ± 0.260 |
| **∆EEV_RCp_** | 0.057 ± 0.321 | 0.097 ± 0.253 | 0.088 ± 0.251 | 0.102 ± 0.275 | 0.098 ± 0.274 | 0.103 ± 0.259 | 0.110 ± 0.278 |
| **∆EEV_RCa_** | 0.042 ± 0.118 | 0.044 ±0.098 | 0.046 ± 0.099 | 0.050 ±0.097 | 0.055 ± 0.102 | 0.051 ± 0.088 | 0.056 ± 0.085 |
| **∆EEV_AB_** | -0.069 ± 0.191 | -0.023 ± 0.171 | -0.019 ± 0.170 | 0.009 ± 0.168 | 0.028 ± 0.151 | 0.022 ±0.160 | 0.031 ± 0.142 |
| **∆EEV_CW_** | 0.026 ± 0.460 | 0.120 ± 0.328 | 0.115 ± 0.346 | 0.147 ± 0.358 | 0.181 ± 0.381 | 0.174 ±0.331 | 0.194 ± 0.337 |
| **Ti (s)** | 2,126 ± 0,779 | 1,911 ± 0,480 | 1,954 ± 0,591 | 1,968 ± 0,735 | 1,893 ± 0,551 | 1,954 ± 0,648 | 1,933 ± 0,660 |
| **Te (s)** | 2,539 ± 0,864 | 2,549 ± 1,062 | 2,243 ± 0,757 | 2,453 ± 0,794 | 2,304 ± 0,778 | 2,377 ± 0,642 | 2,331 ± 0,868 |
| **Ttot(s)** | 4,733 ± 1,484 | 4,414 ± 1,319 | 4,195 ± 1,227 | 4,358 ± 1,350 | 4,226 ± 1,164 | 4,314 ± 1,200 | 4,295 ± 1,404 |

Data shown as mean ± standard deviation. Dark black numbers represent significant differences when compared with quiet breathing.

**Supplementary table 4**. Effects of **expiratory contraction from residual volume** on inspiratory time (Ti), expiratory time (Te) and total time of respiratory cycle (Ttot), end-inspiratory (EIV) and end-expiratory (EEV) chest wall (CW) and compartmental (pulmonary ribcage [RCp]; abdominal ribcage [RCa] and abdominal [Ab]) volume changes (∆) after first (A), second (B) and third (C) maneuvers.

**A)**

|  | **1º cycle** | **2º cycle** | **3º cycle** | **4º cycle** | **5º cycle** | **6º cycle** | **7º cycle** |
| --- | --- | --- | --- | --- | --- | --- | --- |
| **∆EIV_RCp_** | 0.047 ± 0.220 | 0.021 ± 0.187 | -0.022 ± 0.204 | -0.005 ± 0.190 | -0.028 ± 0.158 | -0.035 ± 0.163 | -0.044 ± 0.151 |
| **∆EIV_RCa_** | 0.021 ± 0.106 | 0.013 ± 0.094 | 0.003 ± 0.092 | 0.013 ± 0.080 | 0.004 ± 0.087 | 0.002 ± 0.081 | 0.001 ± 0.084 |
| **∆EIV_AB_** | -0.043 ± 0.202 | -0.052 ± 0.202 | -0.045 ± 0.233 | -0.011 ± 0.306 | -0.005 ± 0.323 | 0.005 ± 0.298 | 0.009 ± 0.323 |
| **∆EIV_CW_** | 0.017 ± 0.406 | -0.019 ±0.354 | -0.069 ± 0.330 | -0.007 ± 0.350 | -0.035 ± 0.387 | -0.032 ±0.323 | -0.042 ±0.378 |
| **∆EEV_RCp_** | **-0.154 ± 0.125** | **-0.084 ±0.114** | -0.055 ± 0.139 | -0.045 ±0.137 | -0.037 ± 0.130 | -0.047 ± 0.117 | -0.042 ±0.081 |
| **∆EEV_RCa_** | -0.035 ± 0.061 | -0.015 ± 0.054 | -0.009 ± 0.042 | -0.003 ± 0.053 | 0.008 ± 0.061 | 0.002 ± 0.065 | -0.009 ±0.060 |
| **∆EEV_AB_** | **-0.156 ± 0.204** | **-0.078 ±0.195** | -0.059 ±0.225 | -0.038 ± 0.241 | 0.014 ±0.247 | 0.014 ± 0.272 | 0.011 ± 0.284 |
| **∆EEV_CW_** | **-0.353 ± 0.288** | **-0.181 ± 0.246** | **-0.128 ±0.248** | -0.091 ± 0.258 | -0.016 ±0.297 | -0.033 ± 0.335 | -0.032 ± 0.333 |
| **Ti (s)** | 2,153 **±** 0,690 | 1,982 **±** 0,726 | 1,823 **±** 0,695 | 2,077 **±** 0,767 | 1,982 **±** 0,761 | 1,972 **±** 0,781 | 2,102 **±** 0,718 |
| **Te (s)** | 2,547 **±** 1,095 | 2,282 **±** 0,619 | 2,453 **±** 1,029 | 2,039 **±** 0,664 | 2,198 **±** 0,712 | 2,172 **±** 0,769 | 2,580 **±** 1,242 |
| **Ttot(s)** | 4,761 **±** 1,464 | 4,263 **±** 1,149 | 4,253 **±** 1,363 | 4,170 **±** 1,249 | 4,219 **±** 1,337 | 4,233 **±** 1,485 | 4,684 **±** 1,645 |

Data shown as mean ± standard deviation. Dark black numbers represent significant differences when compared with quiet breathing.

**B)**

|  | **1º cycle** | **2º cycle** | **3º cycle** | **4º cycle** | **5º cycle** | **6º cycle** | **7º cycle** |
| --- | --- | --- | --- | --- | --- | --- | --- |
| **∆EIV_RCp_** | 0.029 ± 0.223 | -0.002 ± 0.193 | -0.020 ± 0.175 | -0.043 ± 0.159 | -0.062 ± 0.166 | -0.056 ± 0.146 | -0.043 ± 0.155 |
| **∆EIV_RCa_** | 0.050 ± 0.103 | 0.024 ± 0.087 | 0.016 ±0.084 | 0.009 ± 0.079 | 0.007 ± 0.083 | 0.009 ± 0.076 | 0.012 ± 0.084 |
| **∆EIV_AB_** | 0.070 ± 0.284 | 0.035 ± 0.247 | 0.039 ± 0.259 | 0.039 ± 0.250 | 0.021 ± 0.259 | 0.047 ± 0.248 | 0.063 ± 0.278 |
| **∆EIV_CW_** | 0.142 ± 0.425 | 0.054 ± 0.313 | 0.031 ± 0.343 | 0.002 ± 0.311 | -0.036 ± 0.359 | -0.003 ± 0.314 | 0.031 ±0.364 |
| **∆EEV_RCp_** | **-0.154 ± 0.141** | **-0.120 ±0.100** | **-0.071 ±0.105** | **-0.067 ± 0.106** | **-0.082 ± 0.087** | -0.066 ± 0.106 | -0.057 ± 0.102 |
| **∆EEV_RCa_** | **-0.021 ± 0.054** | **-0.005 ± 0.060** | 0.010 ± 0.066 | 0.007 ± 0.067 | 0.007 ± 0.067 | 0.013 ± 0.075 | 0.014 ± 0.077 |
| **∆EEV_AB_** | -0.086 ± 0.237 | -0.036 ± 0.208 | 0.015 ± 0.224 | 0.003 ± 0.217 | 0.021 ± 0.224 | 0.040 ± 0.233 | 0.052 ± 0.248 |
| **∆EEV_CW_** | **-0.262 ± 0.291** | **-0.168 ± 0.236** | -0.048 ± 0.274 | -0.059 ± 0.250 | -0.058 ±0.276 | -0.013 ± 0.287 | 0.007 ± 0.319 |
| **Ti (s)** | 2,165 **±** 0,728 | 2,170 **±** 0,666 | 2,077 **±** 0,774 | 2,047 **±** 0,585 | 1,918 **±** 0,647 | 2,188 **±** 1,100 | 2,051 **±** 0,885 |
| **Te (s)** | 2,320 **±** 0,669 | 2,251 **±** 0,606 | 2,384 **±** 0,784 | 2,237 **±** 0,690 | 2,249 **±** 0,649 | 2,263 **±** 0,728 | 2,367 **±** 0,580 |
| **Ttot(s)** | 4,602 **±** 1,406 | 4,419 **±** 1,093 | 4,491 **±** 1,467 | 4,321 **±** 1,074 | 4,209 **±** 1,180 | 4,465 **±** 1,390 | 4,398 **±** 1,336 |

Data shown as mean ± standard deviation. Dark black numbers represent significant differences when compared with quiet breathing.

**C)**

|  | **1º cycle** | **2º cycle** | **3º cycle** | **4º cycle** | **5º cycle** | **6º cycle** | **7º cycle** |
| --- | --- | --- | --- | --- | --- | --- | --- |
| **∆EIV_RCp_** | 0.021 ± 0.260 | -0.021 ± 0.208 | -0.058 ± 0.218 | -0.056 ± 0.199 | -0.112 ± 0.195 | -0.098 ± 0.202 | -0.111 ± 0.167 |
| **∆EIV_RCa_** | 0.032 ± 0.136 | 0.018 ± 0.110 | 0.012 ± 0.115 | 0.014 ±0.118 | -0.002 ±0.106 | 0.004 ±0.116 | 0.009 ± 0.114 |
| **∆EIV_AB_** | 0.042 ± 0.260 | 0.031 ± 0.293 | 0.039 ±0.292 | 0.038 ± 0.288 | 0.032 ± 0.271 | 0.036 ± 0.269 | 0.032 ± 0.268 |
| **∆EIV_CW_** | 0.086 ± 0.435 | 0.024 ± 0.396 | -0.007 ± 0.415 | -0.012 ± 0.404 | -0.088 ±0.371 | -0.063 ± 0.408 | -0.087 ± 0.376 |
| **∆EEV_RCp_** | **-0.139 ± 0.167** | **-0.114 ± 0.132** | **-0.107 ± 0.131** | **-0.096 ±0.130** | **-0.102 ± 0.137** | **-0.094 ± 0.120** | **-0.088 ±0.116** |
| **∆EEV_RCa_** | **-0.012 ± 0.086** | **-0.011 ± 0.078** | 0.000 ± 0.085 | 0.004 ± 0.089 | 0.011 ± 0.093 | 0.013 ± 0.089 | 0.010 ± 0.095 |
| **∆EEV_AB_** | -0.064 ± 0.257 | -0.028 ± 0.257 | -0.003 ±0.245 | 0.023 ±0.256 | 0.054 ± 0.270 | 0.054 ± 0.258 | 0.043 ± 0.249 |
| **∆EEV_CW_** | **-0.211 ± 0.364** | **-0.154 ± 0.315** | -0.113 ± 0.292 | -0.069 ±0.328 | -0.034 ± 0.362 | -0.027 ±0.327 | -0.035 ± 0.321 |
| **Ti (s)** | 1,988 **±** 0,583 | 2,135 **±** 0,719 | 2,033 **±** 0,708 | 2,035 **±** 0,752 | 1,811 **±** 0,672 | 1,907 **±** 0,634 | 2,007 **±** 0,698 |
| **Te (s)** | 2,282 **±** 0,565 | 2,196 **±** 0,590 | 2,384 **±** 0,818 | 2,202 **±** 0,742 | 2,269 **±** 0,873 | 2,192 **±** 0,672 | 2,088 **±** 0,650 |
| **Ttot(s)** | 4,296 **±** 1,064 | 4,411 **±** 1,210 | 4,442 **±** 1,318 | 4,297 **±** 1,362 | 4,088 **±** 1,398 | 4,174 **±** 1,236 | 4,177 **±** 1,237 |

Data shown as mean ± standard deviation. Dark black numbers represent significant differences when compared with quiet breathing.
